# Longitudinal profiling of the human gut microbiome reveals temporal and personalized responses to inulin

**DOI:** 10.1002/imo2.70029

**Published:** 2025-06-15

**Authors:** Lu Wu, Hong‐Bin Liu, Xu‐Wen Wang, Zi‐Ning Tao, Ze‐Peng Qu, Chao‐Bi Lei, Yang‐Yu Liu, Lei Dai

**Affiliations:** ^1^ State Key Laboratory of Quantitative Synthetic Biology, Shenzhen Institute of Synthetic Biology Shenzhen Institutes of Advanced Technology, Chinese Academy of Sciences Shenzhen China; ^2^ Channing Division of Network Medicine, Department of Medicine Brigham and Women's Hospital and Harvard Medical School Boston Massachusetts USA; ^3^ College of Life Sciences Shandong Agricultural University Tai'an China; ^4^ University of Chinese Academy of Sciences Beijing China

## Abstract

- Inulin has profound and personalized impacts on the dynamics of human gut microbiota and short‐chain fatty acids in stool.
- Inulin intake leads to enrichment of *Bifidobacterium adolescentis*, *Bifidobacterium longum*, *Anaerostipes hadrus*, and *Bacteroides xylanisolvens*.

## Graphical abstract

This study monitored gut microbiome changes in healthy volunteers following inulin intervention, revealing dynamic and highly individualized shifts in microbial composition and short‐chain fatty acid production. Using in vitro batch cultures, correlation analysis, and predictive modeling, we explored the personalized microbiome response. Our findings highlight the individualized response of the gut microbiome to prebiotics and the need for precision nutrition.

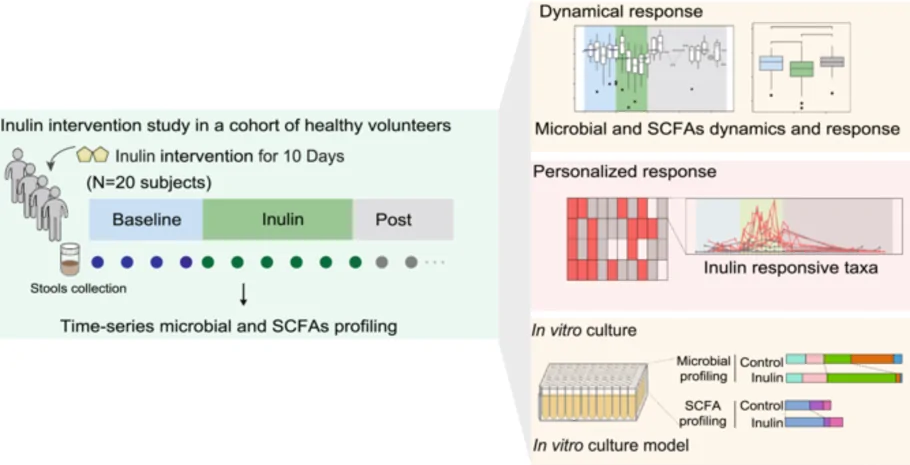

Inulin has been widely used in food applications as a prebiotic and functional food additive. As a beneficial dietary fiber, inulin can modulate the gut microbiota and associated metabolic processes [1]. In the colon, inulin can be fermented into various short‐chain fatty acids (SCFAs) by specific gut microbes, selectively enriching the beneficial microbes, such as *Bifidobacterium* spp. and *Lactobacillus* spp. [2]. These SCFAs have numerous health benefits in maintaining gut health and overall well‐being [3].

However, the human gut microbiota is highly personalized [4]. Recent studies have highlighted the importance of interindividual differences in gut microbiota that can influence the fermentation of dietary fibers [5, 6]. The prebiotic properties of inulin have been extensively studied, focusing on population‐level effects characterized by selective changes in gut microbiota composition [7, 8]. Elucidating interindividual variability in gut microbiota responses to inulin supplementation and determining the characteristics that underlie it are essential first steps toward developing personalized nutritional strategies for targeted microbiome modulation for desirable functionality, encompassing both the enrichment of beneficial microbes and the promotion of SCFA production.

While the gut microbiota's ability to ferment inulin is well‐established [7, 8, 9, 10, 11, 12], the temporal dynamics of microbial succession and associated metabolic outputs during prolonged inulin intervention remain poorly characterized in humans. To address this gap, we conducted a 10‐day longitudinal study tracking microbiome and SCFA profiles in 20 healthy individuals receiving inulin supplementation. We further applied an in vitro batch culture model, correlation analysis, and prediction model to explore the personalized microbiome response. Our study demonstrated that the microbiota responses to inulin intervention in a healthy population exhibit marked subject‐specificity and temporal variability. Therefore, there is a pressing need for personalized assessments of the prebiotic benefits of inulin based on individual human data to guide the design of precision dietary strategies for optimized microbiome outcomes.

## 1 RESULTS AND DISCUSSION

To assess the effects of inulin on the gut microbiota, we conducted a three‐phase study in 20 healthy volunteers (Figure S1): Baseline phase (no intervention) for 1 week; Intervention phase (26 g inulin per day) for 10 days; post‐intervention phase: Follow‐up sampling with at least two stool samples per donor. Donors' lifestyle and clinical characteristics were recorded in Supporting Information (Tables S1, 2). In total, we collected 301 stool samples (with metadata in Table S3), with an average of 14.3 samples per participant. The 16S rRNA V3‐V4 sequencing and GC‐MS were used to characterize the gut microbial composition and SCFA profiles.

**Figure 1:**
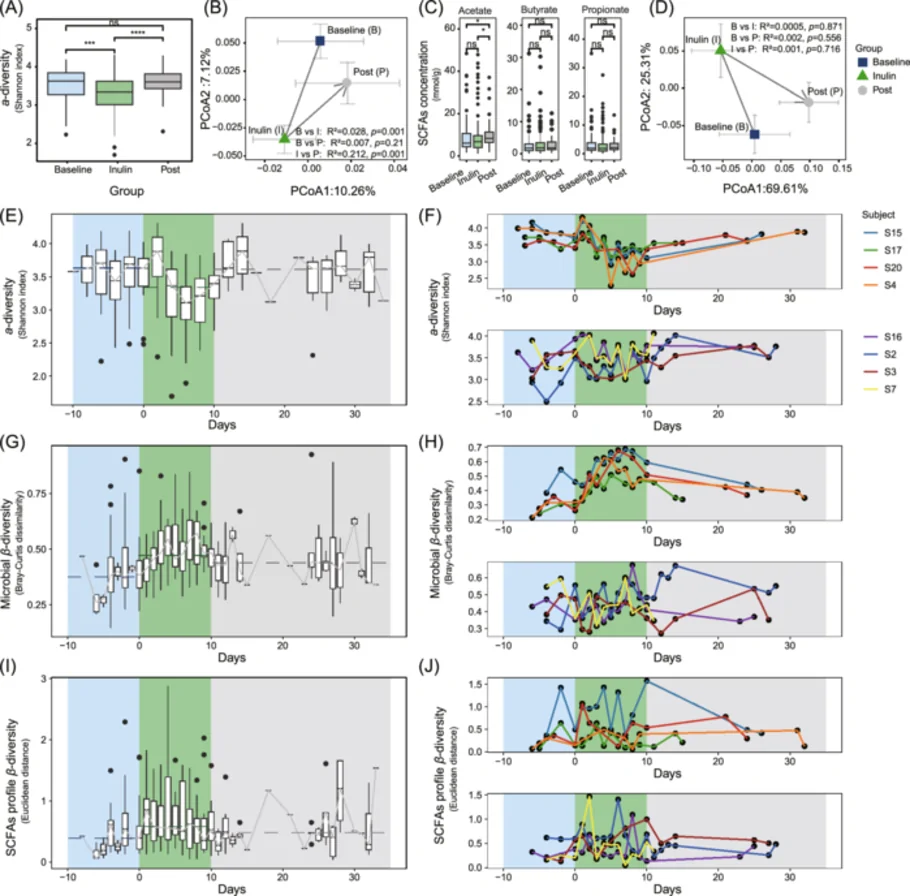
Temporal and individualized response of microbiome composition and short‐chain fatty acid (SCFA) metabolism to inulin intervention. (A) Inulin consumption induced a decrease in microbiota *α*‐diversity (Shannon diversity index) (Mann–Whitney *U* test); (B) PCoA plot with Bray–Curtis dissimilarity revealed that the gut microbial composition was shaped by inulin intervention (PERMANOVA); (C) assessment of average SCFAs production before, during and after the inulin interventions (Mann–Whitney *U* test); (D) PCoA plot with Euclidean distance shown that the gut microbiota SCFAs profiles were shaped by the inulin intervention (PERMANOVA); (E) gut microbiota species diversity (*α*‐diversity, Shannon's Diversity Index) during inulin intervention (Pairwise Wilcox test with Bonferroni *p*‐value adjustment, Baseline vs. Inulin, *p*‐value = 5.25 × 10^−4^, Inulin vs. Post, *p*‐value = 3.48 × 10^−5^). (F) Time series gut microbiota species diversity of eight representative individuals during inulin intervention. Linear mixed‐effects modeling indicated a significant intervention effect in responsive individuals (*p*‐value = 0.0018, F‐top) but not in nonresponsive participants (*p*‐value = 0.7295, F‐bottom). (G) The *β*‐diversity (Bray–Curtis dissimilarities) of each individual's gut microbiota composition to their baseline communities. (H) The time‐series similarity of eight representative individuals' gut microbiota composition to their baseline communities during inulin intervention. (I) Similarity of each individual's stool SCFA metabolic profiles to their baseline SCFAs profiles (*β*‐diversity, Euclidean distance, Pairwise Wilcox test with Bonferroni *p*‐value adjustment, Baseline vs. Inulin, *p*‐value = 3.30 × 10^−11^). (J) The time‐series stool SCFAs profile of 8 representative individuals during inulin intervention (linear mixed‐effects modeling, *p*‐value = 0.0205, I‐top, *p*‐value = 0.0139, I‐bottom). The figure displays three dashed lines representing the median Shannon diversity index/dissimilarities within each phase.

We initially examined the effect of inulin intervention on the gut microbial composition and SCFA profiles at the population level. We found that inulin exerted profound effects on the human gut microbiome. Specifically, it significantly but transiently decreased the community α‐diversity (Figure 1A), and the community composition underwent a significant shift during the inulin intervention. However, during the post‐intervention phase, the community tended to revert to its baseline composition (Figure 1B), consistent with previous studies [11]. While for SCFA metabolism, inulin exhibited a moderate influence on stool SCFA concentration at the population level (Figure 1C), and the inulin‐induced changes in SCFA profile were not significant (Figure 1D).

At the individual level, longitudinal analysis revealed that inulin consumption induced a rapid decrease in the gut microbiota α‐diversity of those individuals within the first 3–4 days (Figure 1E), with significant interindividual variation (Figure S2). Representative examples show responsive (bottom panel) and nonresponsive (top panel) participants (Figure 1F). Linear mixed‐effects (LME) modeling, accounting for interindividual variation, confirmed this pattern (Figure 1F‐TOP), but not in nonresponsive participants (Figure 1F‐BOTTOM). During the post‐intervention phase, eventually all individuals recovered the baseline α‐diversity of their gut microbiota (Figure 1E). These findings suggest that inulin administration can rapidly, uniquely, and transiently alter the α‐diversity of the gut microbiota.

We further assessed the intra‐individual gut microbiome differences induced by inulin. We found that during the intervention phase, individuals' gut microbial compositions significantly differed from their baseline compositions (Figures 1G and S3A). Representative examples demonstrated that inulin intake induced personalized alterations in gut microbial composition (Figure 1H), aligned with the community α‐diversity dynamics. Next, we assessed the intra‐individual differences in the SCFA profile. Inulin can moderately alter the SCFA profiles (Figure 1I), although none of the three SCFAs showed significant alterations at the population level (Figure 1C). Principal coordinate analysis showed noticeable variations among individuals (Figure S3B). Interestingly, regardless of their gut microbiota changes, the LME model revealed‐significant SCFA profile changes across different individuals (Figure 1J). Notably, all donors exhibited marked temporal fluctuations in their SCFA profiles (Figure S3C), potentially influenced by uncontrolled diet during the intervention [13]. Future studies should implement dietary controls (e.g., standardized meals or food tracking) to better define SCFAs dynamics.

Next, we further assessed the personalized response to inulin in terms of the overall community structure (Figure S4A) and SCFA metabolic profile (Figure S4B) changes. We found that 18 out of 20 donors exhibited significant compositional changes. While significant changes were observed in only 9 out of 20 donors for SCFA profiles. Nevertheless, considerable variation among individuals was also noted (Figure S4C,D). We further visualized the baseline communities' gut microbial profiles and the SCFA profile in Figure S4E,F. Distance‐based Redundancy Analysis (dbRDA) revealed that baseline microbiome compositions failed to predict inulin‐induced changes at the group level, either for microbial shifts or SCFA profiles. However, per‐subject dbRDA assessing associations between microbiome changes and SCFA fluctuations during the intervention revealed that distinct subsets of individuals where specific SCFA‐microbiome relationships emerged (Figure S5A). For example, in Subject 11, we observed that microbiome changes could explain propionate and butyrate concentration changes, but not acetate variations (Figure S5B).

Collectively, our longitudinal profiling reveals that inulin exerts profound and personalized effects on the dynamics of the human gut microbial compositions while exhibiting a relatively moderate influence on the stool SCFA profiles, aligning with prior human studies [7, 8, 11, 12]. In addition, we observed interindividual differences in the response patterns to inulin during temporal microbial shifts. Furthermore, paired analysis of gut microbial composition and SCFA profiles revealed a striking dissociation at the population level, changes in microbial compositions did not always coincide with changes in stool SCFA profiles. However, individual‐level analyses identified specific cases where microbial shifts could quantitatively explain SCFA variations.

**Figure 2:**
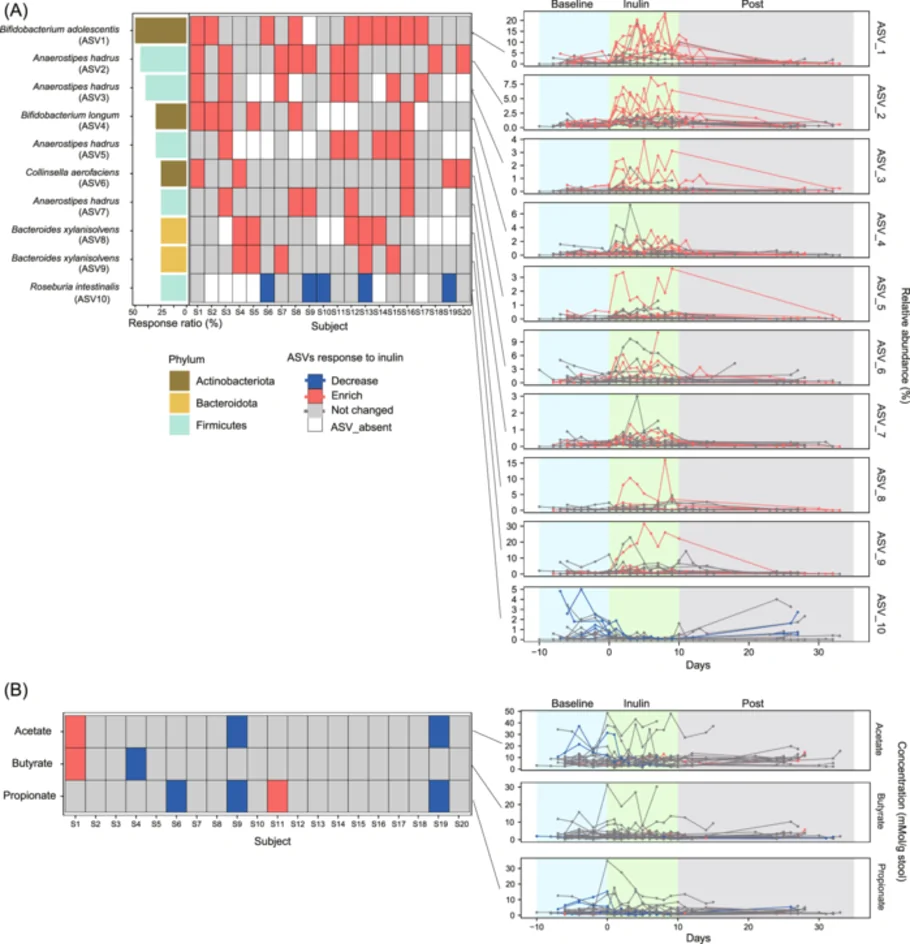
Overview of the taxa and short‐chain fatty acids (SCFAs) responsive to inulin intervention. (A) The distribution pattern of inulin‐responsive taxa among donors. The top 10 ASVs shared by donors are presented here, with significant abundance changes in response to inulin intervention determined using the Mann–Whitney *U* test (*p*‐value < 0.05). Donors with ASVs significantly enriched by inulin are highlighted in red, and those with a significant decrease are highlighted in blue. ASVs that are absent from individuals are shown in white, and ASVs without significant changes are shown in gray. The right pane shows the time‐series relative abundance of the inulin‐responsive taxa. (B) The response of SCFAs to inulin varies among donors. Responders are defined by the Mann–Whitney *U* test (*p*‐value < 0.05) for subjects with significant variance in stool SCFAs concentration during inulin intervention, as marked in red (significantly increased) or blue (significantly decreased), while non‐responders are in gray. The right pane shows the time series concentration of the SCFAs.

We then identified the taxa with differential abundance during the baseline and inulin phases for each individual (Figure 2A). Notably, species with established inulin‐degrading capacity (*Bifidobacterium adolescentis*, *Bifidobacterium longum*, *Anaerostipes hadrus*, *Collinsella aerofaciens*, and *Bacteroides xylanisolvens*) were significantly enriched [14]. We also observed that although present, these inulin degradation features were not enriched by inulin in several donors. Our findings indicated that the putative inulin‐degrading species were enriched in a personalized manner. Ecologically, inulin‐degrading species coexist but compete for inulin, favoring the most efficient metabolizers [5, 10]. We also observed that those species responded rapidly to inulin intake, and the pattern was maintained during the intervention but diminished during the washout phase (Figure S6), which was consistent with our results in Figure 1G. Regarding SCFAs, while the overall SCFA concentrations in stool did not show significant differences at the population level before and during inulin intake (Figure 1D), we observed notable differences at the individual level (Figure S7), 6 out of 20 individuals exhibited a significant response to inulin in terms of the concentration change of SCFAs (Figure 2B). Specifically, the concentrations of acetate, butyrate, and propionate in stool samples showed significant changes within a subset of donors. The temporal SCFA dynamics revealed minimal effects of inulin intervention. It should be noted that the inulin dose used in this study (26 g/day) represents a relatively high intake level compared to the typical recommended dietary consumption. Although our findings demonstrate good short‐term tolerability and transient microbiome impact in healthy participants, we emphasize that this does not necessarily imply safety for long‐term use or in special populations.

To understand and predict personalized microbiome responses to inulin, we integrated in vitro batch culture, correlation analysis, and predictive modeling. In vitro studies have been employed to examine interindividual variability in the gut microbiota's response to various fibers [15, 16]. To validate in vitro‐vivo concordance of gut microbiota responses to inulin, we performed paired in vitro and in vivo comparisons (Figure S8A). In general, inulin significantly shaped the gut microbial composition in vitro (Figure S9A). We observed that inulin in vitro fermentation increased the abundance of *Blautia*, *Anaerostipes*, and *Bifidobacterium* (Figures S8B, S9B). ASVs belonging to *B. adolescentis*, *B. longum*, *A. hadrus*, and *C. aerofaciens* demonstrated congruent enrichment patterns following inulin administration across both in vitro and in vivo models. (Figure S10A). Intriguingly, we consistently observed enrichment of *Blautia* with inulin across donors in vitro, indicating its capability to utilize inulin. Nevertheless, this enrichment was also observed in previous in vitro fermentation studies [15, 17] and in mice [18], but not in human trials [19]. In vitro fermentation of inulin primarily enriched acetate levels while reducing butyrate and propionate levels (Figure S8C, Figure S11). Notably, the inulin‐induced SCFA response pattern for each individual is not always consistent between in vivo and in vitro (Figure S10B). Overall, our findings indicate that in vitro inulin fermentation could reflect personalized compositional changes of gut microbiota in human trials during inulin intervention, but lack correlation with SCFA production. We hypothesize that the divergence in SCFA dynamics between in vitro and in vivo environments may arise from distinct ecological or metabolic constraints, such as contrasts in substrate availability, host absorption processes, and pH gradients. These differences likely drive the selective enrichment of context‐dependent taxa in *vitro* versus in vivo, ultimately shaping SCFA profiles.

Our correlation analyses revealed distinct taxa‐acetate/butyrate associations in vivo and in vitro (Figure S12A). Specifically, the in vivo increase in *A. hadrus* abundance significantly predicted butyrate levels, supporting its known butyrogenic role [17]. A glycan‐degrading genus *Blautia* [14, 17], which was significantly associated with acetate production (Figure S12B), showed increased abundance in 19 of 20 in vitro cultures, paralleling the observed rises in acetate concentrations (Figure S8C). Notably, this *Blautia*‐acetate coupling was absent in vivo, where *Blautia* is neither a dominant nor a responsive taxon. When we calculated the correlation within each donor using the longitudinal data, we further found that the correlation between the responsive species and SCFAs is also personalized within each donor over time (Figure S12C). Donor‐specific taxon‐SCFAs correlations varied by ecological context, underscoring the importance of multi‐factor modeling for predicting gut metabolite dynamics [20].

Inspired by Cremer et al.'s framework [20], which quantitatively estimates the daily amount of fermentation products released by the gut microbiota based on experimental measurements of fermentative metabolism from major gut bacterial strains, we sought to adapt this approach to estimate SCFAs production in our study. We found that while estimated acetate levels did not correlate with measured values (Figure S13A)—likely due to the absence of *Blautia* in Cremer et al.'s characterized strains—the estimated propionate (Figure S13B) and butyrate (Figure S13C) levels showed significant correlations with experimental measurements. This suggests that enrichment of key genera (e.g., *Lachnospiraceae* and *Bifidobacteriaceae*) may drive a substantial fraction of SCFAs changes, particularly for propionate and butyrate. These results support the feasibility of modeling SCFAs dynamics from microbiome composition and biomass.

Taken together, these results demonstrate that while inulin stimulates the growth of inulin‐degrading species, the resulting metabolic outcomes are highly individualized. Microbial shifts do not consistently translate to elevated SCFA concentrations in stool, and taxon‐SCFA correlations themselves show marked interindividual variations. The dual‐layer heterogeneity in both taxon responsiveness and their metabolic associations could explain why reliable prediction of SCFA production requires incorporation of multi‐strain quantitative metabolic data, as demonstrated by Cremer et al.'s framework, rather than relying on single taxon‐metabolite correlations.

## 2 CONCLUSION

Our study demonstrates that inulin induces transient yet personalized gut microbiota shifts, with marked interindividual variation in the enrichment of inulin‐degrading taxa (e.g., *B. adolescentis*, *B. longum*, *A. hadrus*, and *B. xylanisolvens*), while exerting minimal effects on stool SCFA profiles at the population level. Notably, in vitro experiments replicated taxonomic changes but failed to predict in vivo SCFA dynamics, underscoring context‐dependent ecological limitations. Integrating multi‐modal data, our study emphasized the significant variability in individual responses to inulin, suggesting the need for further research to evaluate inulin's desirable functionality from two distinct perspectives: the enrichment of beneficial microbes and the promotion of SCFAs production. Future research should prioritize large‐scale human cohorts with standardized dietary controls to bridge the gap between microbial modulation and clinically relevant metabolic outcomes.

## 3 METHODS

The comprehensive methodology, encompassing study design, experimental procedures, and data analysis and visualization, is available in the Supporting Information.

## AUTHOR CONTRIBUTIONS

**Lu Wu**: Funding acquisition; writing—original draft; writing—review and editing; visualization; validation; methodology; data curation; resources; project administration. **Hong‐Bin Liu**: Writing—original draft; writing—review and editing; visualization; methodology; data curation; project administration. **Xu‐Wen Wang**: Methodology; writing—review and editing. **Zi‐Ning Tao**: Methodology; project administration. **Ze‐Peng Qu**: Methodology. **Chao‐Bi Lei**: Methodology. **Yang‐Yu Liu**: Writing—review and editing; funding acquisition; supervision. **Lei Dai**: Supervision; writing—review and editing; funding acquisition.

## CONFLICT OF INTEREST STATEMENT

All authors declare no conflicts of interest.

## ETHICS STATEMENT

This study was approved by the Shenzhen Institute of Advanced Technology, Chinese Academy of Sciences (SIAT‐IRB‐210515‐H0564). All participants gave written informed consent before participating in the study.

## Supporting information

The online version contains supplementary figures and tables available.

**Figure S1.** In vivo inulin intervention experiment design.
**Figure S2.** Time series of the α‐diversity for each donor's gut microbiome during inulin intervention.
**Figure S3.** Personalized gut microbiome signature during inulin intervention.
**Figure S4.** Personalized alterations in microbial composition and SCFA metabolism after inulin interventions.
**Figure S5.** Individual‐level correlations between the microbial composition and the inulin‐induced microbial response.
**Figure S6.** Time‐series relative abundance of inulin‐responsive species dynamics in each donor.
**Figure S7.** The change of SCFAs in the gut during inulin intervention was personalized.
**Figure S8.** *In vitro* inulin batch fermentation reflects personalized changes in microbial composition and SCFA production, but it does not always align with the changes in vivo.
**Figure S9.** Inulin intervention reshaped the gut microbiota composition in vitro.
**Figure S10.** Compositional changes and SCFA production correlation between in vitro and in vivo with inulin intervention.
**Figure S11.** Personalized SCFA changes in vitro with inulin intervention.
**Figure S12.** Correlation between microbiome and SCFA.
**Figure S13.** Performance of SCFA production predictions using experimentally derived metabolite exchange rates.

**Table S1.** Descriptive characteristics of participants.
**Table S2.** Individual‐level participant characteristics.
**Table S3.** Microbiome sample metadata.

## Data Availability

The data that support the findings of this study are openly available in PRJEB81240 at https://www.ebi.ac.uk/ena/browser/view/PRJEB81240, reference number PRJEB81240. All sequencing data generated in this study are available from the European Nucleotide Archive (ENA) under study accession number PRJEB81240 (https://www.ebi.ac.uk/ena/browser/view/PRJEB81240, with metadata in Tables S1, S2, and S3). Supplementary materials (methods, figures, tables, metadata, graphical abstract, slides, videos, Chinese translated version, and update materials) may be found in the online DOI or iMetaOmics http://www.imeta.science/imetaomics/.
