# Supplementary material for: Longitudinal profiling of the human gut microbiome reveals temporal and personalized responses to inulin
Source: IMetaOmics. 2025 Jun 15;2(3):e70029. doi: 10.1002/imo2.70029 (PMC12379954; doi:10.1002/imo2.70029)
Supplement: Supplementary file 1 — Figure S1. In vivo inulin intervention experiment design. Figure S2. Time series of the α‐diversity for each donor's gut microbiome during inulin intervention. Figure S3. Personalized gut microbiome signature during inulin intervention. Figure S4. Personalized alterations in microbial composition and SCFA metabolism after inulin interventions. Figure S5. Individual‐level correlations between the microbial composition and the inulin‐induced microbial response. Figure S6. Time‐series relative abundance of inulin‐responsive species dynamics in each donor. Figure S7. The change of SCFAs in the gut during inulin intervention was personalized. Figure S8. In vitro inulin batch fermentation reflects personalized changes in microbial composition and SCFA production, but it does not always align with the changes in vivo. Figure S9. Inulin intervention reshaped the gut microbiota composition in vitro. Figure S10. Compositional changes and SCFA production correlation between in vitro and in vivo with inulin intervention. Figure S11. Personalized SCFA changes in vitro with inulin intervention. Figure S12. Correlation between microbiome and SCFA. Figure S13. Performance of SCFA production predictions using experimentally derived metabolite exchange rates. [file IMO2-2-e70029-s001.docx]

**Supporting Information for**

**Longitudinal profiling of the human gut microbiome reveals temporal and personalized responses to inulin**

**Running title: Individualized gut microbiome dynamics during inulin treatment**

Lu Wu^1#^, Hong-Bin Liu^1#^, Xu-Wen Wang^2^, Zi-Ning Tao^1,3^, Ze-Peng Qu^1^, Chao-Bi Lei^1^, Yang-Yu Liu^2*^, Lei Dai^1,4*^

^1^CAS Key Laboratory of Quantitative Engineering Biology, Shenzhen Institute of Synthetic Biology, Shenzhen Institute of Advanced Technology, Chinese Academy of Sciences, Shenzhen 518055, China.

^2^Channing Division of Network Medicine, Department of Medicine, Brigham and Women’s Hospital and Harvard Medical School, Boston, MA, 02115, USA.

^3^College of Life Sciences, Shandong Agricultural University, Tai’an 271000, China.

^4^University of Chinese Academy of Sciences, Beijing 100049, China.

^#^These authors contributed equally: Lu Wu, Hong-Bin Liu

^*^Correspondence: [yyl@channing.harvard.edu](mailto:yyl@channing.harvard.edu) (Yang-Yu Liu), [lei.dai@siat.ac.cn](mailto:lei.dai@siat.ac.cn) (Lei Dai)

**Methods**

**Study participants.** Self-reported healthy volunteers without a history of antibiotics exposure in the past three months were recruited from the Shenzhen Institute of Advanced Technology, Chinese Academy of Sciences. All participants gave written, informed consent before participating in the study. Participants ranged in age from 24 to 35, with a median age of 29.6. This study was approved by the Shenzhen Institute of Advanced Technology, Chinese Academy of Sciences (SIAT-IRB-210515-H0564).

**Study design and inulin intake.** During the first week of this study, each volunteer collected four stool samples every other day, which refer to four baseline data. During the second week, volunteers underwent a 10-day inulin intervention phase with 26 g inulin (Orafti HP, BENEO-Orafti). The dose was selected based on BMI-adjusted tolerability data from prior studies [1] and administered in two divided servings (13 g each, morning and evening with warm water) to optimize gastrointestinal tolerance [2]. At least six stool samples were collected on separate days during this phase, referred to as four inulin samples. In the study's third phase, volunteers stopped the inulin intake but continued to collect at least two samples on separate days, referred to as post data.

***In vitro* inulin fermentation.** Stool samples were collected from volunteers and immediately transferred into the anaerobic workstation (85% N_2_, 10% H_2,_ and 5% CO_2_, COY specify model). For each volunteer, 10 g of stool from the last sample collected during the baseline phase was suspended into 50 mL 20% glycerol (in sterile phosphate-buffered saline, with 0.1% L-cysteine hydrochloride). The samples were homogenized by vortexing and then filtered with sterile nylon mesh to remove large particles in fecal matter. Aliquots of the suspension were placed in sterile cryogenic vials and frozen at -80°C for long-term storage until use. 20 μL stool suspension was inoculated into 980 μL of medium in 96-well plates (PCR-96-SG-C, Axygen), with 5 g of inulin (Orafti HP, BENEO-Orafti, 5% w/v) added. The cultures were then statically incubated at 37°C in the anaerobic workstation for 24 h. Cultures without inulin were incubated as a control. Each incubation was done in two replicates. The medium used for *in vitro* culture was modified from previous studies [3], which comprises: peptone water (2.0 g /L, CM0009, Thermo Fisher), yeast extract (2.0 g /L, LP0021B, Thermo Fisher), L-cysteine hydrochloride (1 g/ L), Tween 80 (2 mL/L), hemin (5 mg/L), vitamin K1(10 μL/L), NaCl (1.0 g /L), K_2_HPO_4_ (0.4 g/L), KH_2_PO_4_ (0.4 g/L), MgSO_4_⋅7H_2_O (0.1 g/L), CaCl_2_⋅2H_2_O (0.1 g/L), NaHCO_3_ (4 g/L), porcine gastric mucin (4 g/L, M2378, Sigma-Aldrich), sodium cholate (0.25 g/L) and sodium chenodeoxycholate (0.25 g/L). After 24 h’s fermentation, samples were centrifuged, the supernatant was stored at -80°C for Gas Chromatography-Mass Spectrometry (GC-MS) analysis, and the pellets were stored at -80°C with a plastic seal until DNA extraction.

**16S rRNA gene amplicon sequencing and compositional analysis.** Stool sample DNA was extracted using the QIAamp Power Fecal Pro DNA Kit (Qiagen, 51804) according to manufacturer’s instructions. *In vitro* fermentation sample DNA was extracted using the DNeasy UltraClean 96 Microbial Kit (Qiagen, 10196-4) according to manufacturer’s instructions. 16S rRNA amplicon library preparation was performed using an automated Tecan Freedom EVO 200 protocol. The V_3_-V_4_ region of the 16S rRNA gene was amplified using primers 341F 5’-CCTACGGGNGGCWGCAG -3’ and 805R 5’-GACTACHVGGGTATCTAATCC-3’ with barcodes. Libraries were further pooled together at equal molar ratios and sequenced by Illumina NovaSeq (250 bp paired-end reads) at Novogene Technology (Tianjin, China). 16S rRNA amplicon sequencing data were analyzed by QIIME2 (version 2020.2) [4]. Primers of the raw sequence data were cut with Cutadapt (via q2-cutadapt) [5]. Quality control was performed by DADA2 (via q2-dada2) [6]. All amplicon sequence variants (ASVs) from DADA2 were used to construct a phylogenic tree with fasttree (via q2-phylogeny) [7]. The ASVs were assigned to taxonomy with naïve Bayes classifier (via q2-feature-classifier) [4] against the SILVA database (SILVA_132_SSURef_Nr99) [8]. The ASV table was normalized, and rare ASVs (all features with a total abundance of less than ten and present in only a single sample) were filtered out.

**Quantification of short-chain fatty acids (SCFAs) concentration by GC-MS.** The SCFAs were analyzed by GC-MS. For the stool sample extraction, 0.05 g of frozen stool sample or 50 μL fermentation supernatant was mixed with 300 µL of pure internal standard (final concentration 20 μg/mL). After adding 1.0 mm diameter zirconia/silica beads (BioSpec, Bartlesville), the stool was homogenized for 20 s under 6500 rpm three times, then incubated at 4°C with shaking for 30 min, followed by centrifugation for 30 min at 13,000 × *g*. For the fermentation sample extraction, 50 μL fermentation supernatant was mixed with 300 µL of pure internal standard (final concentration 20 μg/mL) before use. Following extraction of internal standard-containing stool supernatant or fermentation supernatant with anhydrous diethyl ether, the SCFA extract was accurately transferred into a glass insert in a GC vial and capped tightly after adding 5µL of N, O-bis (trimethyl-silyl) -trifluoroacetamide and vortexed for 5s. The mixture was kept in the GC vial and incubated at room temperature (22°C) overnight (or over 8 h) before loading to GC/MS. The analysis of acetic, propionic, and butyric acids was performed by Agilent 8890/7000D triple quadrupole GC/MS equipped with a capillary HP-5 ms capillary column (30 m × 0.25 mm × 0.25 µm film thickness) (Agilent Technologies). The analyses were quantified in the selected ion monitoring (SIM) mode using the target ion and confirmed by confirmative ions. The integrated areas for all SCFAs were normalized with the internal standard and quantified with the standard curve, as previously described [9].

**Data visualization.** $\alpha$-diversity of the community was calculated on the Shannon index. The composition of the microbiota was analyzed using PCoA with the Bray-Curtis dissimilarity metric applied to the ASV compositional profile, while the SCFA metabolic profiles were analyzed using Euclidean distance. Similarities among groups in PCoA was determined by permutational multivariate analysis of variance (PERMANOVA, Adonis test) [10], with 999 permutations used to test the significance. These analyses were conducted using the vegan package (version 2.6-4) [11]. Non-parametric Mann-Whitney *U* tests were used to conduct pairwise comparisons between two groups, and Pairwise Wilcox test with Bonferroni *p*-value adjustment were used to compare three groups [12]. Distance-based redundancy analysis (dbRDA) was performed to assess whether baseline microbial community profile variations (Bray-Curtis dissimilarity) explain inulin-induced (i) microbiome shifts (Bray-Curtis dissimilarity) or (ii) SCFA profiles changes (Euclidean distance). To evaluate personalized responses, we additionally conducted per-subject dbRDA, modeling within-individual associations between microbiome and SCFA changes over time. P values of less than 0.05 were considered statistically significant, as indicated in the figures (ns, not significant, **p*-value < 0.05, ***p*-value < 0.01, ****p*-value < 0.001, *****p*-value < 0.0001). Data analysis and plotting were performed in R version 4.1.2 and R studio (version 2022.12.0 + 353) using the packages dplyr, ggpubr, and vegan.

**Supplementary Figures**


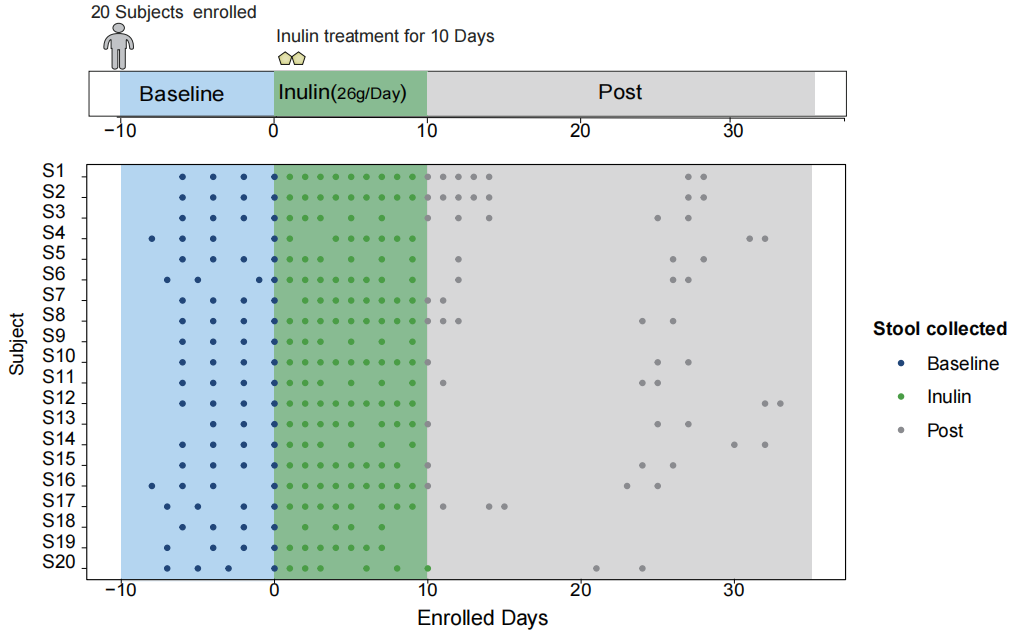


**Figure S1 *In vivo* inulin intervention experiment design.** This study investigated personalized gut microbial responses to inulin intervention *in vivo* using longitudinal data from 20 participants. Before the intervention, each participant collected at least four baseline stool samples. During the 10-day intervention, they were encouraged to collect samples at least every other day, followed by a post-intervention observation phase. Stool samples were collected at baseline, during, and after inulin administration, and plotted for each volunteer.


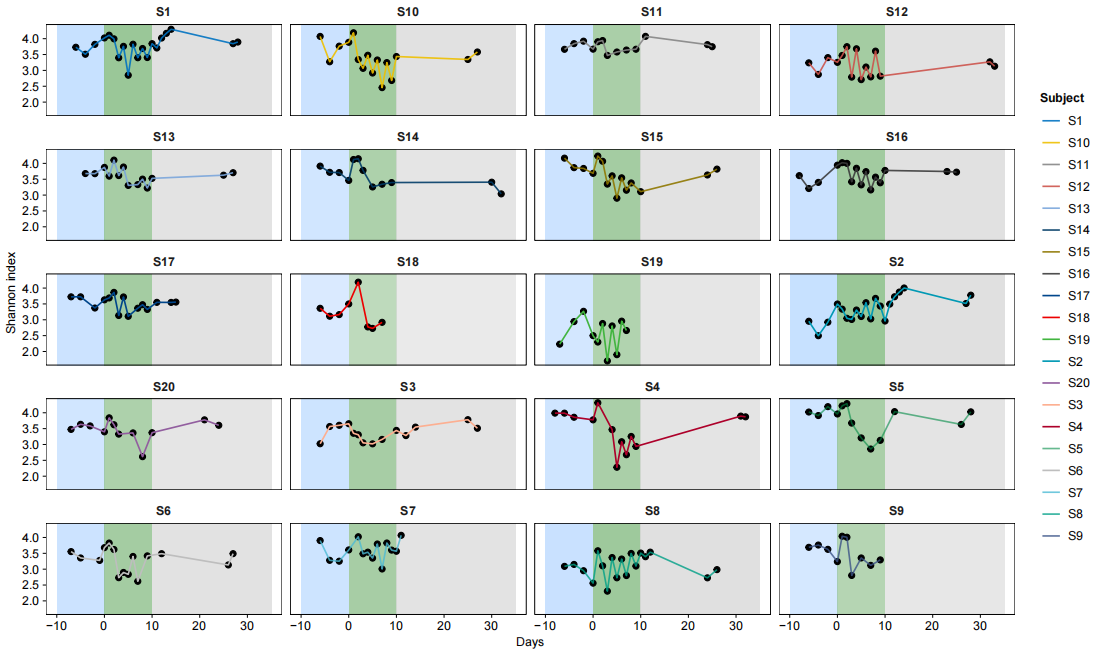


**Figure S2 Time series of the** $\boldsymbol{\alpha}$**-diversity for each volunteer’s gut microbiome during inulin intervention.** Each line shows one volunteer's gut microbiome $\alpha$-diversity (Shannon index) during inulin treatment.


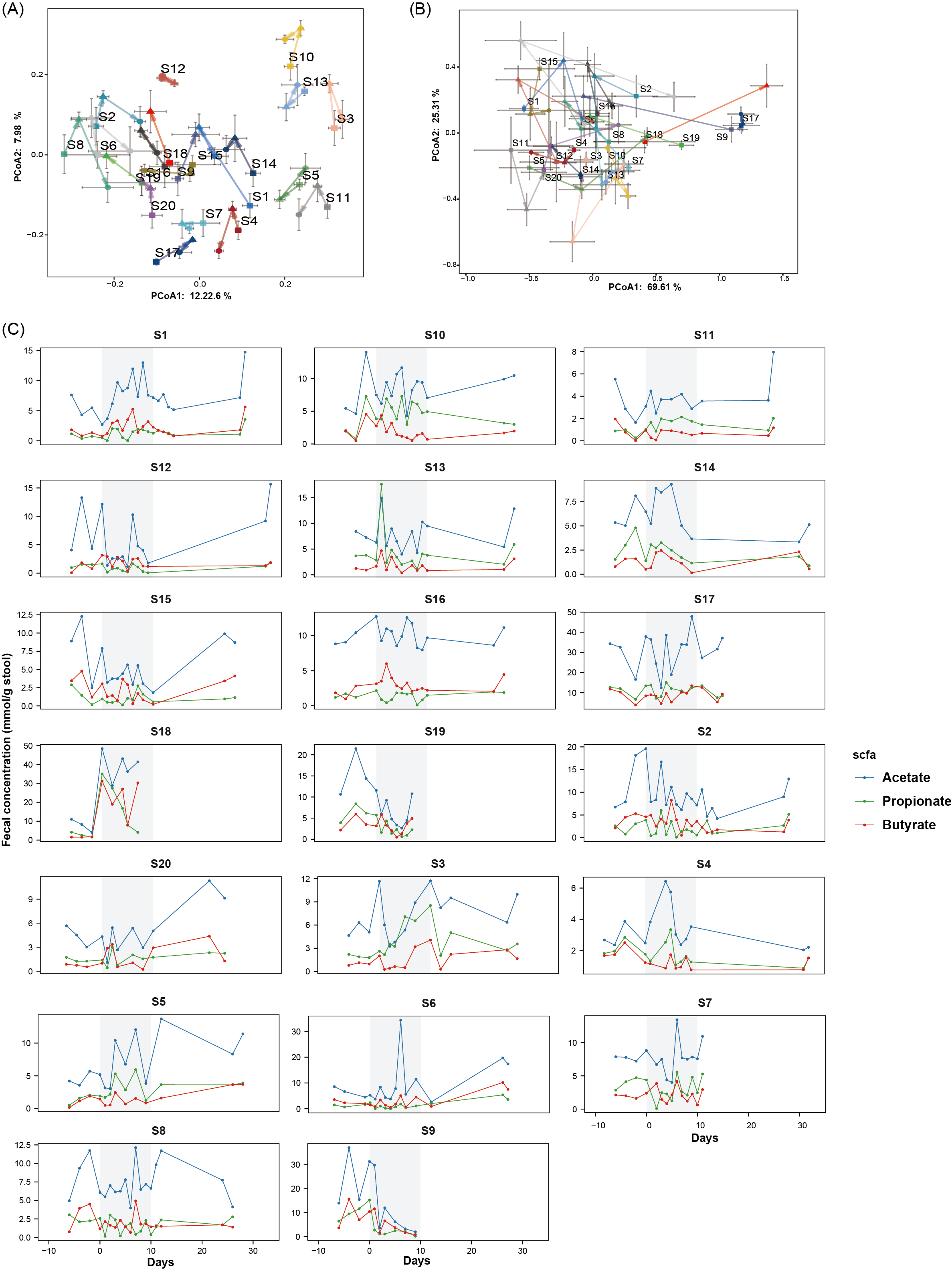


**Figure S3 Personalized gut microbiome signature during inulin intervention.** (A) PCoA plot (Bray-Curtis dissimilarities) illustrating the gut microbiota composition of 20 volunteers, categorized into different groups during inulin intervention, revealed that the personalized characteristics of each donor's gut microbiota had a dominant influence over the effects of the inulin intervention. Symbols represent the average position of each donor within their respective group, while error bars depict the standard error. (B) PCoA plot (Euclidean distance) illustrating the gut SCFA profile of 20 donors, categorized into different groups during inulin intervention, revealed substantial dynamics in the SCFA profiles for each donor throughout the inulin intervention. Symbols represent the average position of each donor within their respective group, while error bars depict the standard error. (C) The time series concentration changes for three SCFAs (Acetate, Butyrate, and Propionate) in stool for each donor during the inulin intervention.


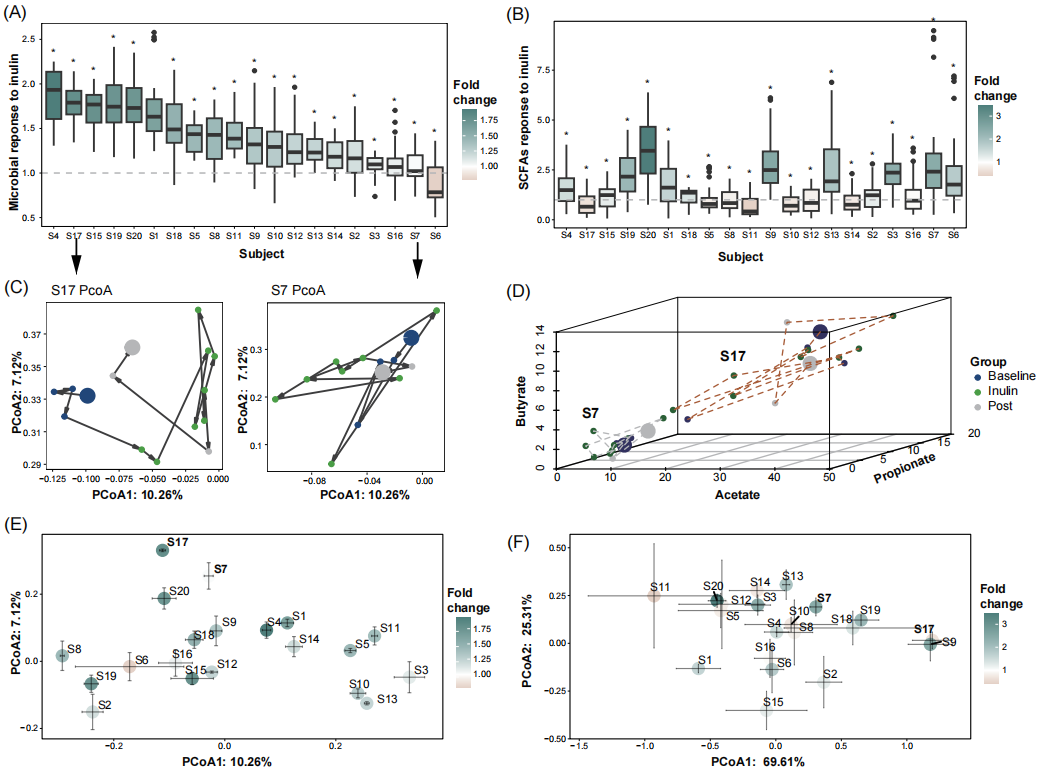


**Figure S4 Personalized alterations in microbial composition and SCFA metabolism after inulin interventions.** (A) The rank of inulin-induced microbial composition response for each individual was evaluated by calculating the fold change in dissimilarity between the pair-wise Bray-Curtis dissimilarity for both inter-group samples (baseline and inulin phases) and within-group samples (baseline samples) (Mann-Whitney *U* test, *p*-value < 0.05). (B) The inulin-induced SCFA metabolic response for each individual was evaluated by calculating the fold change in distance between the pair-wise Euclidean distance for both inter-group samples (baseline and inulin phases) and within-group samples (baseline samples) (Mann-Whitney *U* test, *p*-value < 0.05). Subjects were ordered according to their microbial response ranking from panel A. (C) PCoA showing the personalized trajectory of microbial composition (C, Bray-Curtis dissimilarity) during the inulin intervention. The gut microbial composition of subject S17 demonstrated a significant response to inulin, whereas subject S7 did not exhibit any notable change. (D) 3D trajectories of individual SCFA profiles (Acetate [x], Propionate [y], Butyrate [z] in mM) during the intervention. While Subject S17 (left) showed visible SCFA fluctuations and S7 (right) appeared stable, PERMANOVA analysis found neither reached statistical significance (S17: *p*-value = 0.996; S7: *p*-value = 0.638). The baseline is represented by blue dots, inulin by green dots, and post-intervention by gray dots. The start and end time points are shown with a larger dot size, and the dots are connected to indicate the temporal trajectory of the microbiota for this subject. (E) PCoA plots based on the Bray-Curtis dissimilarity of the composition profiles of baseline communities. Color of the point showing the average composition changes (A) with inulin. (F) PCoA plots based on the Euclidean distance of the SCFA profiles of baseline communities. Color of the point showing the average SCFAs changes (B) with inulin. Asterisks (*) represent the individuals who exhibited significant changes in microbial composition (A) and stool SCFA profiles (B) during inulin intervention, as determined by the Mann-Whitney *U* test (*p*-value < 0.05).


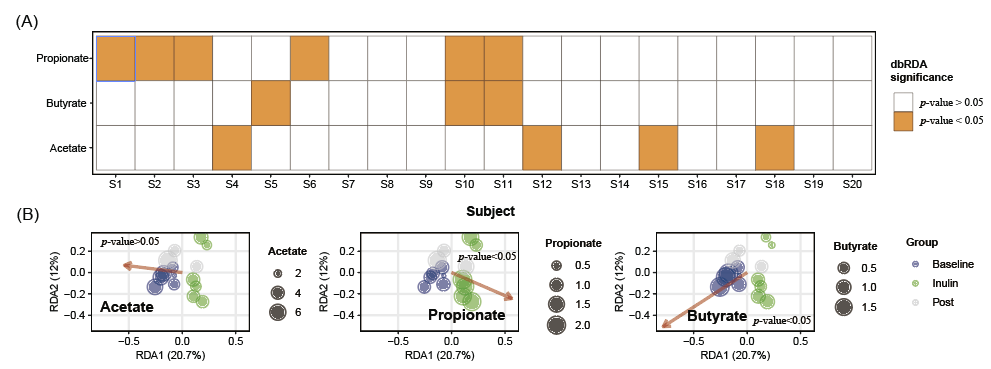


**Figure S5 Individual-level correlations between the microbial composition and the inulin-induced microbial response.** (A) Correlation distribution between microbial composition and inulin-induced SCFA changes across all donors. Brown highlights indicate volunteers showing significant associations (dbRDA, *p*-value < 0.05) between microbial community shifts and SCFA profile changes. (B) PCoA ordination of volunteer S11's longitudinal microbiome variation (Bray-Curtis dissimilarity) during inulin intervention, with SCFA vectors (Acetate, Propionate, and Butyrate) from dbRDA showing their explanatory power for compositional variation. The length and direction of vectors represent the strength and nature of associations between SCFA changes and microbiome shifts. The points represent individual samples, colored by collection phase and sized by total SCFAs concentration.


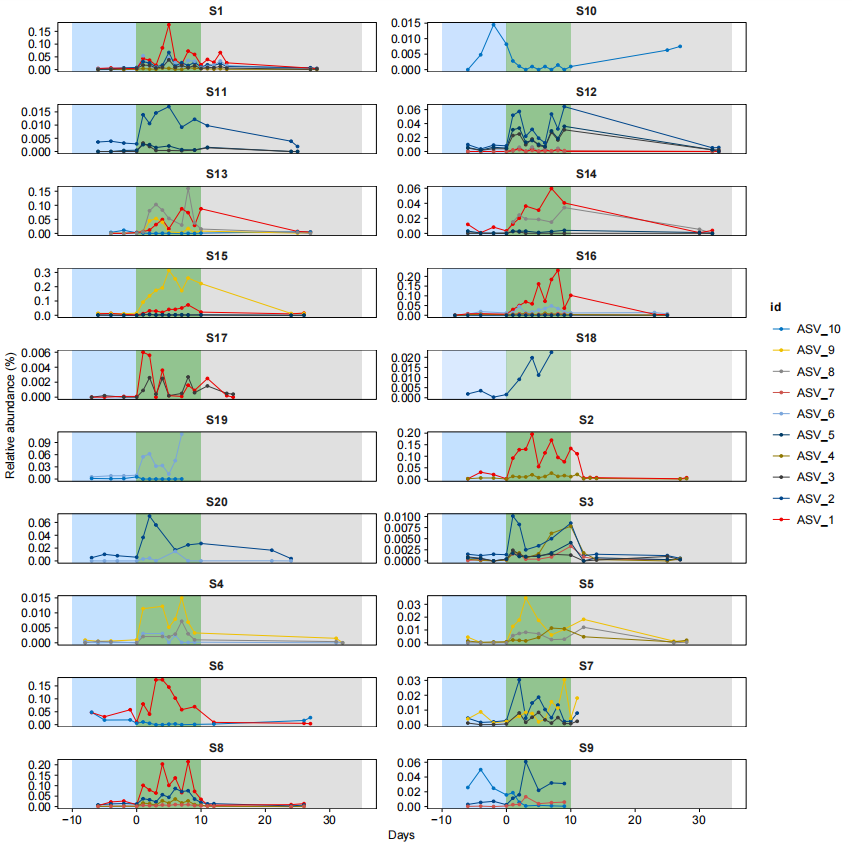


**Figure S6 Time-series relative abundance of inulin-responsive species dynamics in each volunteer.** Shown are the top 10 most responsive ASVs in Figure 2. Only ASVs with significant abundance changes (*p-*value < 0.05) during inulin intervention are displayed.


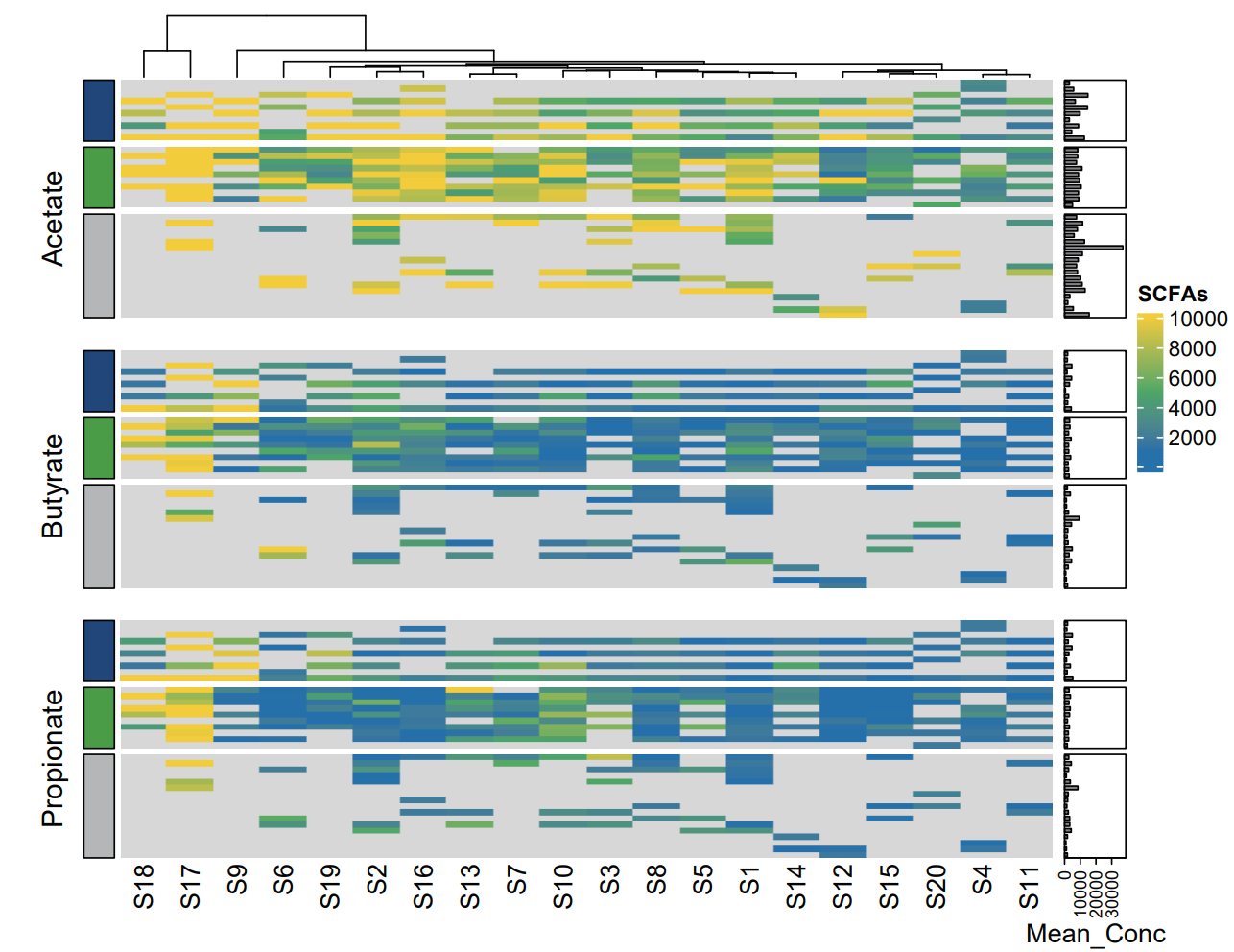


**Figure S7 The change of SCFAs in the gut during inulin intervention was personalized.** A heatmap illustrated the fluctuations in SCFA concentration throughout the inulin intervention for each volunteer. The left panel displayed the types of SCFAs (Acetate, Butyrate, and Propionate) and groups (Baseline, Inulin, and Post) for each stool sample in bar graphs. The barplot in the right panel presented the time-series concentration of SCFAs (average concentration per day).


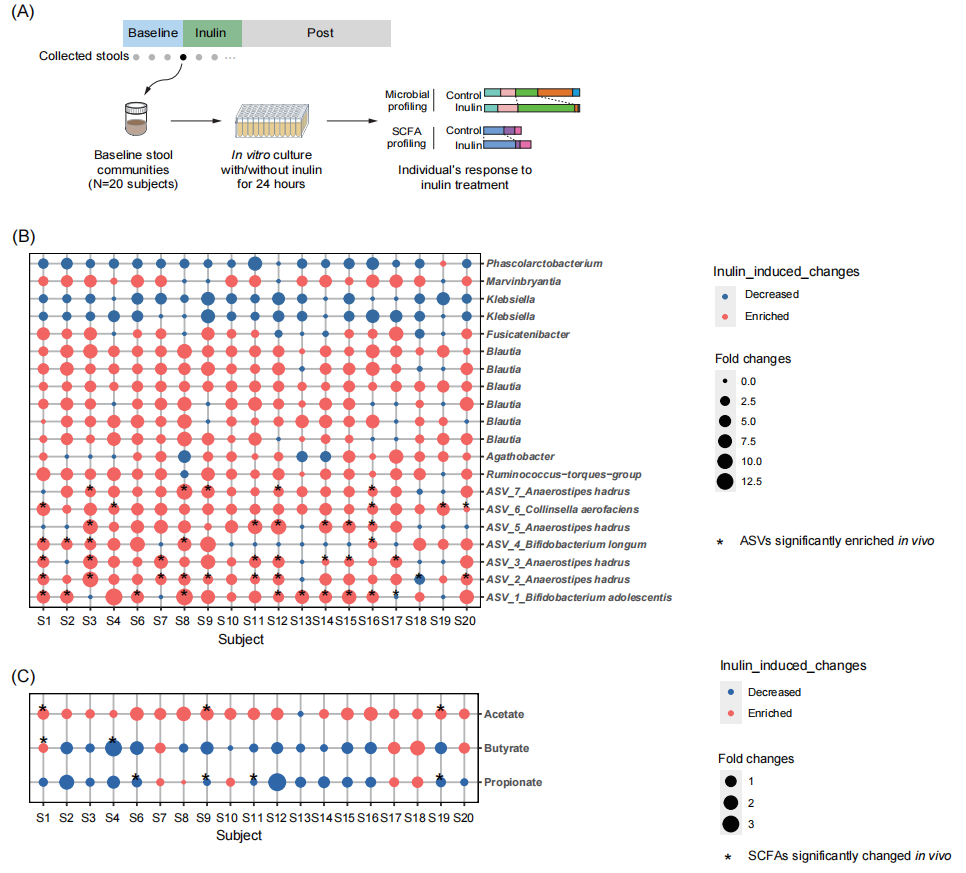


**Figure S8 *In vitro* inulin batch fermentation reflects personalized changes in microbial composition and SCFA production, but it does not always align with the changes *in vivo*.** (A) The design of the *in vitro* inulin batch fermentation experiment workflow. Stool samples utilized in the *in vitro* assays were collected from baseline stool samples of each volunteer prior to inulin intervention. (B) The features exhibiting top abundance change (Log_2_ Inulin/Control) in response to inulin intervention *in vitro*; (C) SCFA production change (Log_2_ Inulin/Control) *in vitro*. * represents the ASVs/SCFAs that were significantly changed during inulin intervention in that specific individual *in vivo*.


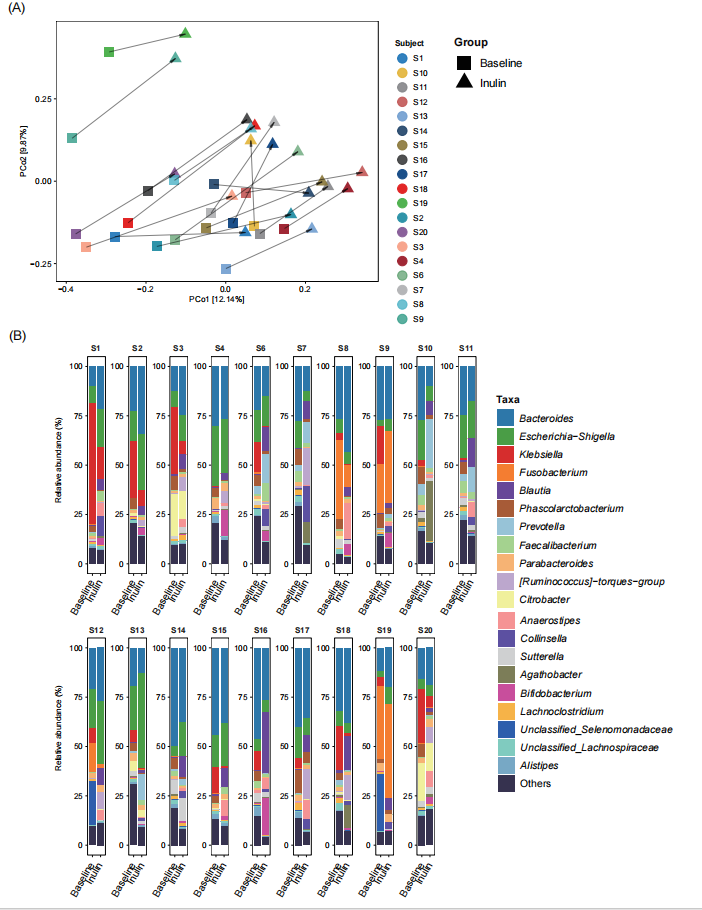


**Figure S9 Inulin intervention reshaped the gut microbiota composition *in vitro*.** (A) The PCoA plot, utilizing Bray-Curtis dissimilarity, illustrated that inulin altered the gut microbiota composition consistently among volunteers *in vitro*. (B) A barplot demonstrated the shift in gut microbiota composition during the inulin intervention *in vitro*.


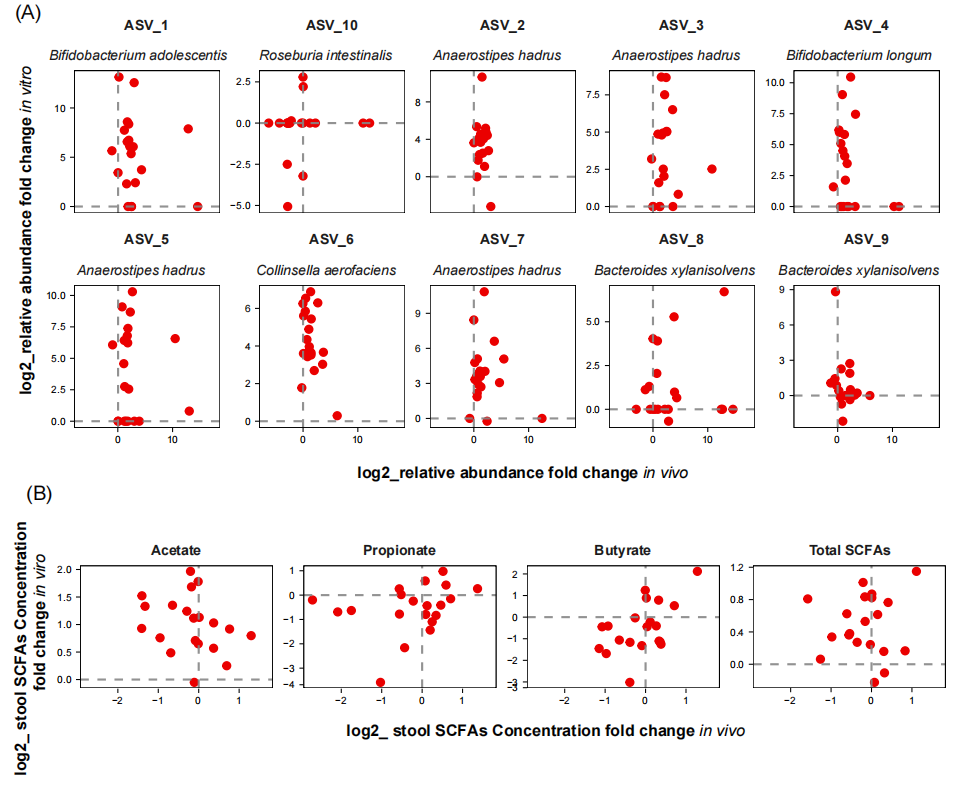


**Figure S10 Compositional changes and SCFA production correlation between *in vitro* and *in vivo* with inulin intervention.** (A) The correlation between the relative abundance change of inulin-responsive species *in vivo* (Log_2_ Inulin/Baseline) and in the MiPro medium *in vitro* (Log2 Inulin/Control); (B) The correlation between the stool SCFA concentration change *in vivo* (Log_2_ Inulin/Baseline) and in the MiPro medium *in vitro* (Log_2_ Inulin/Control).


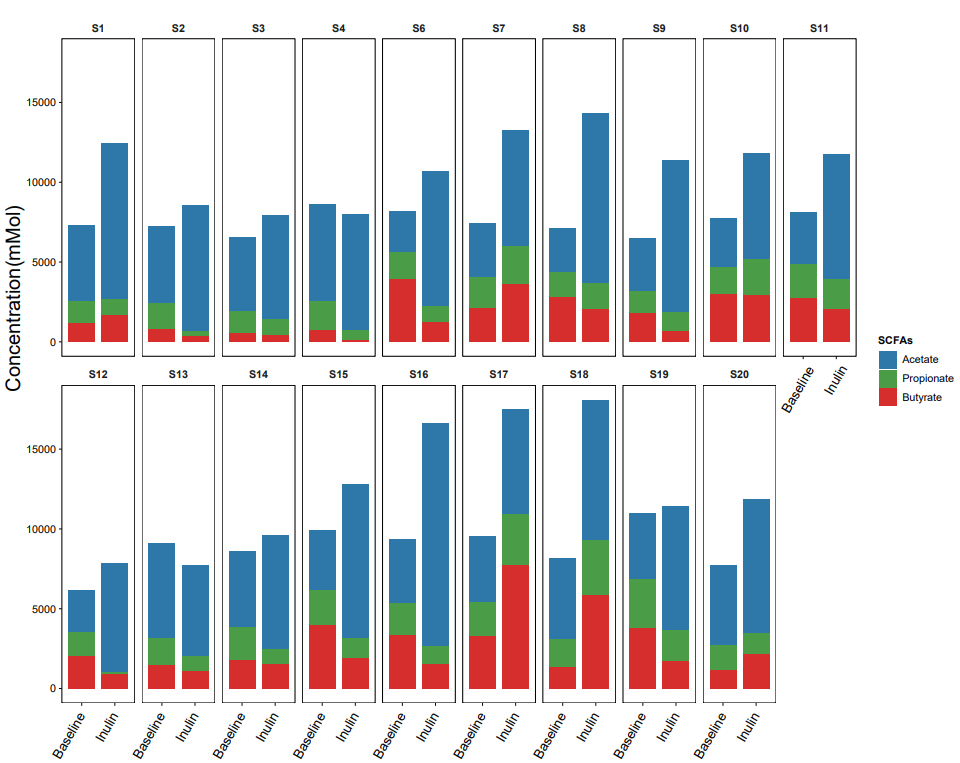


**Figure S11 Personalized SCFA changes *in vitro* with inulin intervention.** A barplot demonstrated the shift in gut microbiota SCFA profile during the inulin intervention *in vitro*.


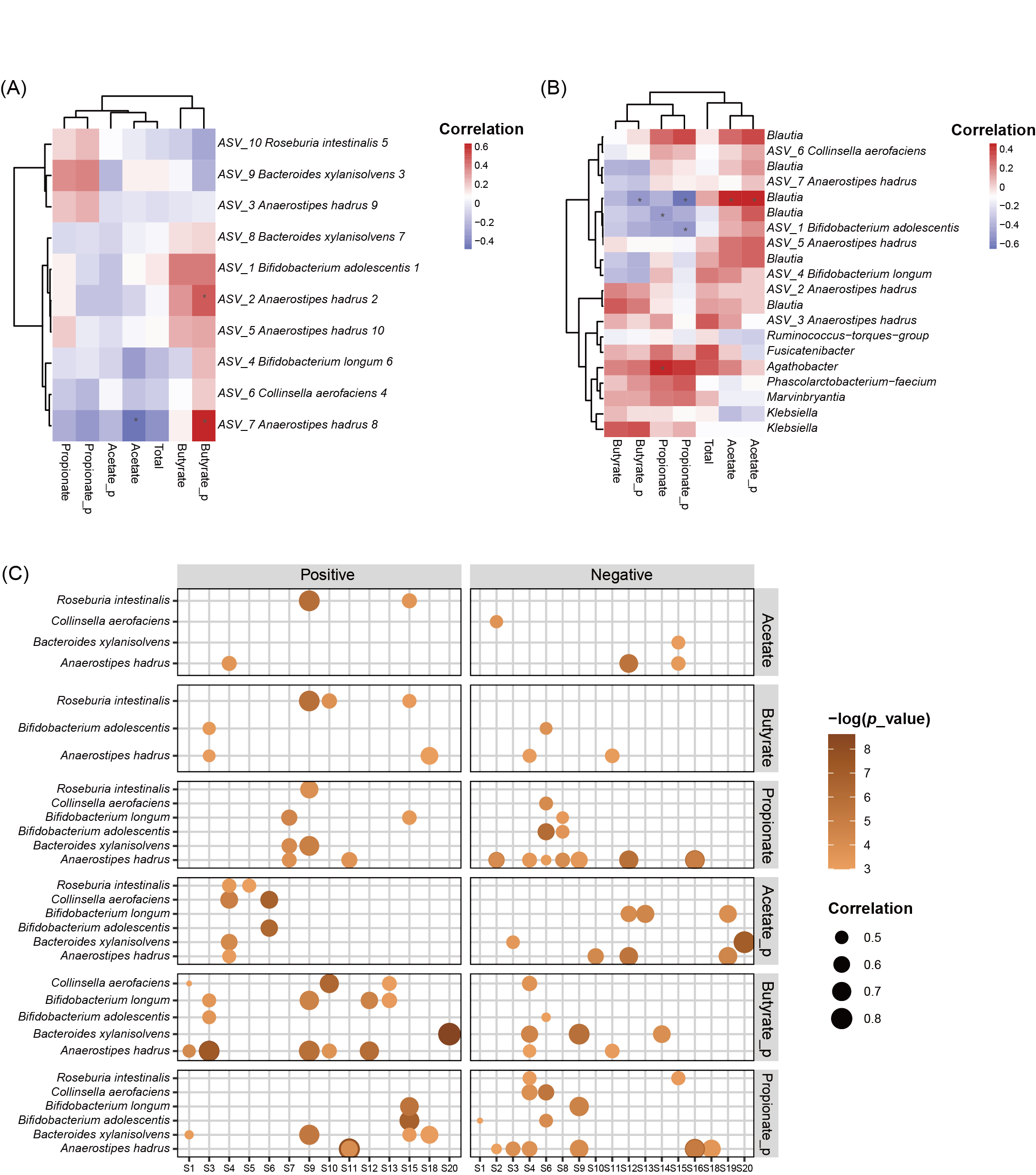


**Figure S12 Correlation between microbiome and SCFA.** (A-B) Spearman correlations between inulin-responsive taxa and SCFA changes *in vivo* (A) and *in vitro* (B), showing absolute concentration changes (Acetate, Butyrate, Propionate) and proportional changes (Acetate_p, Butyrate_p, Propionate_p). (C) Personalized longitudinal correlations *in vivo* visualized through bubble plots, displaying within-subject associations (Spearman’s correlation, *p*-value < 0.05) between SCFA concentrations/proportions and species-level taxonomy assignments (bubble size: correlation strength; color: *p*-value).


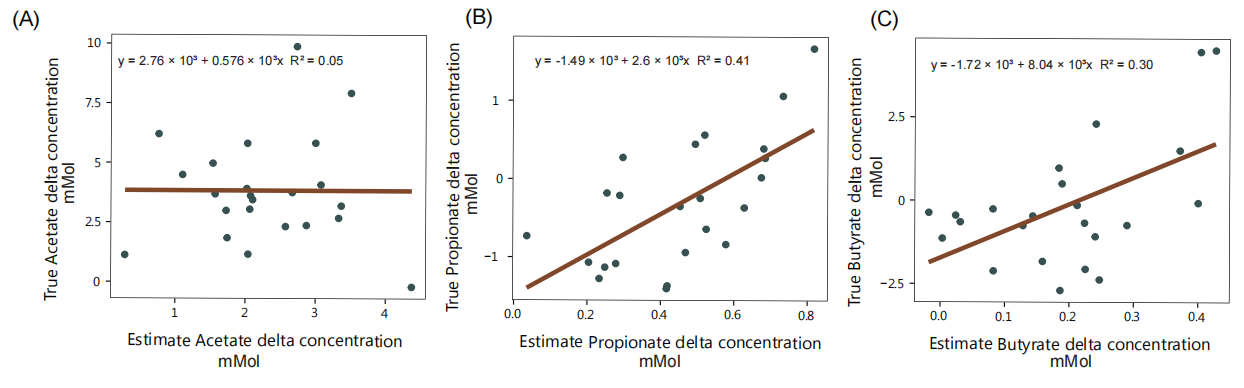


**Figure S13 Performance of SCFA production predictions using experimentally derived metabolite exchange rates.** Predicted daily SCFA production was estimated by integrating in vitro biomass measurements (OD_600_), taxonomic composition changes during intervention, and strain-specific fermentation product excretion rates from Cremer et al*.*'s characterization of 16 abundant gut species. Comparisons of predicted versus measured stool SCFA concentrations changes revealed poor correlation for Acetate (A**,** Pearson correlation, *r* = -0.001426677, *p*-value = 0.9948) but significant correlations for Propionate (B**,** Pearson correlation, *r* = 0.6397428, *p*-value = 0.001012) and Butyrate (C**,** Pearson correlation, *r* = 0.5455729, *p*-value = 0.007088) across volunteers.

**REFERENCE**

1. François, Isabelle E. J. A., Olivier Lescroart, Wim S. Veraverbeke, Karen Windey, Kristin Verbeke, Willem F. Broekaert. 2014. “Tolerance and the effect of high doses of wheat bran extract, containing arabinoxylan-oligosaccharides, and oligofructose on faecal output: a double-blind, randomised, placebo-controlled, cross-over trial.” *Journal of Nutritional Science* 3: e49. <https://doi.org/10.1017/jns.2014.52>

2. Coussement, Paul. 1999. “Inulin and oligofructose: safe intakes and legal status.” *The Journal of Nutrition* 129: 1412S-1417S. <https://doi.org/10.1093/jn/129.7.1412S>

3. Li, Leyuan, Elias Abou-Samra, Zhibin Ning, Xu Zhang, Janice Mayne, Janet Wang, Kai Cheng, Krystal Walker, Alain Stintzi, Daniel Figeys. 2019. “An in vitro model maintaining taxon-specific functional activities of the gut microbiome.” *Nature Communications* 10: 4146. <https://doi.org/10.1038/s41467-019-12087-8>

4. Nicholas, A. Bokulich, Benjamin D. Kaehler, Jai Ram Rideout, Matthew Dillon, Evan Bolyen, Rob Knight, Gavin A. Huttley, J. Gregory Caporaso. 2018. “Optimizing taxonomic classification of marker-gene amplicon sequences with QIIME 2's q2-feature-classifier plugin.” *Microbiome* 6: 90. <https://doi.org/10.1186/s40168-018-0470-z>

5. Martin, Marcel. 2011. “Cutadapt removes adapter sequences from high-throughput sequencing reads.” *EMBnet. journal* 17(1): pp–10. <https://doi.org/doi:10.14806/ej.17.1.200>

6. Benjamin, J Callahan, Paul J McMurdie, Michael J Rosen, Andrew W Han, Amy Jo A Johnson, Susan P Holmes. 2016. “DADA2: High-resolution sample inference from Illumina amplicon data.” *Nature Methods* 13: 581-583. <https://doi.org/10.1038/nmeth.3869>

7. Morgan, N. Price, Paramvir S. Dehal,Adam P. Arkin. 2010. “FastTree 2--approximately maximum-likelihood trees for large alignments.” *PLoS One* 5: e9490. <https://doi.org/10.1371/journal.pone.0009490>

8. Christian, Quast, Elmar Pruesse, Pelin Yilmaz, Jan Gerken, Timmy Schweer, Pablo Yarza, Jörg Peplies, Frank Oliver Glöckner. 2013. “The SILVA ribosomal RNA gene database project: improved data processing and web-based tools.” *Nucleic Acids Research* 41: D590-596. <https://doi.org/10.1093/nar/gks1219>

9. Cai Jingwei, Jingtao Zhang, Yuan Tian, Limin Zhang, Emmanuel Hatzakis, Kristopher W. Krausz, Philip B. Smith, Frank J. Gonzalez, Andrew D. Patterson. 2017. “Orthogonal comparison of GC-MS and (1)H NMR Spectroscopy for short chain fatty acid quantitation.” *Analytical Chemistry* 89: 7900-7906. <https://doi.org/10.1021/acs.analchem.7b00848>

10. Clarke, K. Robert. 1993. “Non‐parametric multivariate analyses of changes in community structure.” *Australian Journal of Ecology* 18: 26. <https://doi.org/10.1111/j.1442-9993.1993.tb00438.x>

11. Dixon, Philip. 2003. “VEGAN, a package of R functions for community ecology.” *Journal of Vegetation Science* 14.6: 3. <https://doi.org/10.1111/j.1654-1103.2003.tb02228.x>

12. Nicola, Segata, Jacques Izard, Levi Waldron, Dirk Gevers, Larisa Miropolsky, Wendy S Garrett, Curtis Huttenhower. 2011. “Metagenomic biomarker discovery and explanation.” *Genome Biology* 12: R60. <https://doi.org/10.1186/gb-2011-12-6-r60>
